# The “SES NXT” digital intervention for children of relationship dissolution: Study protocol for a randomized controlled trial study

**DOI:** 10.1016/j.invent.2024.100797

**Published:** 2024-12-12

**Authors:** Camilla S. Øverup, Daniel B. Johnsen, Martin Skriver, Søren Sander, Theis Lange, Gert Martin Hald

**Affiliations:** a Department of Public Health, University Of Copenhagen, Øster Farimagsgade 5, 1353 København K, Denmark; b Samarbejde efter Skilsmisse, ApS Aldersrogade 6A, 2 sal, 2100 København Ø, Denmark; c School of Psychology, Deakin University, Australia

**Keywords:** Children, Parental relationship dissolution, Divorce, Digital intervention

## Abstract

Parental relationship dissolution is among the most prevalent life crises for youths and is associated with both short- and long-term intra- and interpersonal struggles. Extant support programs tend to be in-person and in a group format. However, the structure and personnel needed for these programs make them costly to implement, less accessible, and difficult to scale. Digital interventions may present a suitable alternative. The current study examines the effectiveness of an online psycho-social intervention for children who have experienced parental relationship dissolution in Denmark, using a two-arm, parallel-group, randomized controlled trial study design. Families are recruited through Danish municipalities and the Danish Agency of Family Law and randomly assigned to the intervention group or wait-list control group. Individuals are assessed at baseline, 4 weeks, and 12 weeks post-baseline; parents complete questionnaires on behalf of their children aged 3–10, while youth aged 11–17 complete the questionnaires themselves. The primary study outcomes are 1) emotional problems symptoms, as measured by the Strength and Difficulty Questionnaire (SDQ), 2) mental well-being related difficulties, represented by the SDQ-Total scale score, and 3) impact of problems on daily life, as assessed by the SDQ-Impact scale score, at 12-weeks post-baseline. The data will be analyzed using a generalized estimating equation, accounting for non-independence of data (nesting of children within a family). The present study will contribute to the extant knowledge about the effectiveness of digital interventions for youths experiencing parental relationship dissolution and contribute to a cost-effective evidence-based scalable psychological help for a population who needs it.

## Highlights

• Parental relationship dissolution is linked with intra- and interpersonal struggles.
• Protocol for a randomized controlled trial study with a waitlist control group
• Recruitment through Danish municipalities and the Danish Agency of Family Law
• Program may provide more flexible and cost-effective treatment alternatives.

Parental relationship dissolution is among the most prevalent life crises for youths (Boring et al., 2015). In Denmark, 22 % of all youths living with their parents are split between two households, and annually 24.000 youths transition from living in a nuclear family to other types of living arrangements (Statistics Denmark, 2018). Research shows that parental relationship dissolution is negatively associated with youths' mental well-being, quality of life, and social and academic development (Amato, 2001, Amato, 2014; Amato and Keith, 1991; Kunz, 2001), including learning difficulties, lower self-esteem, poorer relationships with parents and friends, and symptoms of depression, anxiety, behavioral problems, and risky health behaviors, both in the short term and in the long term (Auersperg et al., 2019; Fallesen et al., 2023; Kunz, 2001; Størksen et al., 2005). Consequently, there is a need for effective, scalable, and accessible interventions for youths.

Many post-divorce interventions have been developed to help adults and children cope with a divorce. Those aimed at parents have tended to yield positive intra-personal outcomes for the adults themselves as well as improved parental cooperation and co-parenting (e.g., Saini and Corrente, 2024). These programs also appear to have some downstream positive effects in terms of improved child adjustment post-divorce (Fackrell et al., 2011; Saini and Corrente, 2024). Several preventive interventions have also been developed directly targeting youths experiencing parental relationship dissolution, such as Children's Support Group (CSG) (Stolberg and Garrison, 1985), Children of Divorce Intervention Program (CODIP) (Pedro-Carroll and Cowen, 1985), and Kids in Divorce Situations (KIDS) (Pelleboer-Gunnink et al., 2015). These programs are often based on psychoeducational and cognitive-behavioral principles and are facilitated in a group format led by a coach or therapist. Studies of these programs have yielded positive results in improving internalizing and externalizing problems (Haine et al., 2003; Pruett and Barker, 2010). However, the structure and personnel needed for these programs make them costly to implement, less accessible, and difficult to scale. More generally, meta-analyses and narrative reviews suggest that the effects of these programs are varied. Poli et al. (2017) found that, overall, group-based programs for children with divorced or separated parents appear to yield positive outcomes in terms of self/individual level variables (e.g., self-esteem and anxiety), family (e.g., parent-child relations) and interpersonal (e.g., social skills) relations, school (e.g., academic performance) and behavior (e.g., behavioral problems) (Poli et al., 2017). Stathakos and Roehrle (2003) showed that intervention programs for youths experiencing relationship dissolution were efficacious in reducing anxiety and depression symptoms and improving academic behavior and performance, with a medium average effect size (*d* = 0.43). However, only around half of the studies were judged to be of high methodological quality and those studies showed a lower average effect (*d* = 0.32) (Stathakos and Roehrle, 2003). Interestingly, they also showed that the interventions were most effective when the interventions were implemented early (i.e., within 30 months of the divorce), and when the interventions were less comprehensive (i.e., fewer and shorter sessions) (Stathakos and Roehrle, 2003). Conversely, a more recent meta-analysis by Herrero et al. (2023) demonstrated overall small and statistically non-significant effects on children's internalizing (e.g., depression, anxiety) and externalizing (i.e., aggressiveness, behavioral problems, learning problems, and negative peer relationships) symptoms, but medium to large and robust effects in terms of children's self-esteem and divorce adjustment (Hedge's *g* = 0.56–0.70). Moreover, the analyses also suggested some heterogeneity in effects, and some evidence of indirect effects, such that studies with greater changes in internalizing symptoms reported greater changes in externalizing symptoms.

Digital interventions are often more affordable, easier to scale, and have the ability to be updated and expanded (Kazdin and Blase, 2011). Digital interventions may also be preferred by people experiencing relationship dissolution, as they can be more convenient, accessible anytime anywhere, and help reduce stigmatization (Bowers et al., 2011; Greenberg et al., 2019). Recent research shows that digital interventions for distressed couples and adults undergoing relationship dissolution and divorce yield moderate to large effects (Doss et al., 2016; Hald et al., 2020a). However, there is a dearth of research-based digital interventions aimed at youths (Bowers et al., 2014; Haine et al., 2003). To our knowledge, there is only one digital intervention aimed directly at youths of divorced parents, namely the Children of Divorce-Coping with Divorce (CoD-CoD) (Boring et al., 2015), with another under development (O'Hara et al., 2023). The CoD-CoD intervention consists of five self-paced modules, adapted from evidence-based cognitive–behavioral coping programs for adolescents (aged 11–16 years) (Boring et al., 2015). Evaluations of the CoD-CoD interventions showed that the intervention was successful in reducing youth-reported mental health problems and adjustment problems among youths experiencing parental relationship dissolution and also among youths experiencing high levels of parental conflict (Boring et al., 2015; O'Hara et al., 2022). Although the CoD-CoD has demonstrated promising results, the CoD-CoD has only been developed for and tested among older youths. The intervention currently under development (O'Hara et al., 2023) tests 3 digital modules focused on teaching coping skills (i.e., reappraisal, distraction, and relaxation) to help children cope with a high-conflict parental divorce. However, similar to CoD-CoD, the intervention only targets a small subset of children, specifically those aged 9 to 12. As relationship dissolution affects youths across different ages (Pedro-Carroll, 2005), it is imperative to develop interventions that can encompass and be scaled to multiple age groups.

The current study evaluates the efficacy of a newly developed digital intervention program for youths experiencing parental relationship dissolution, named Cooperation After Divorce – NXT (abbreviated ‘SES NXT’ as Cooperation After Divorce = ‘Samarbejde Efter Skilsmisse’ in Danish). The SES NXT intervention is a brief, modular and age-adjusted intervention developed in line with the most prevailing theory in divorce research, namely the Divorce-Stress-Adjustment Perspective (DSAP) (Amato, 2000). Divorce is understood as a process that exposes the child to several stress factors, which can have a cumulatively negative effect on the child over time. These stress factors may include parental conflict, difficulty communicating with parents, navigating new home environments, and perhaps new ‘bonus’ families (Amato, 2014; Ottosen et al., 2018). The extent to which the youth is affected by the parental relationship dissolution, and the long-term consequences thereof, is related to the balance between these stressors, the youth's psychological make-up, and the youth's access to key protective factors, such as good coping skills (O'Hara et al., 2019), good child-parent relations and communication (Gunter Castleton, 2020; Seijo et al., 2016), and the interpretation and meaning made of the dissolution (e.g., loss, gain, change, healing) (Brand et al., 2017).

Thus, the objective of this present study is to examine whether the SES NXT intervention is effective in improving mental and physical health and well-being among youth and children, aged 3 to 17 years, who have experienced parental relationship dissolution. The study employs a randomized controlled trial (RCT) design, with assessments at baseline (T1), 4 weeks post-baseline (T2), and 12 weeks post-baseline (T3), comparing an intervention group, who will receive unlimited access to the SES NXT intervention, to a waitlist control group, who will not. We have developed the following primary study hypotheses:

H1) The intervention group will report a significantly lower SDQ emotional symptoms score than the control group at 12 weeks follow-up (T3).
H2) The intervention group will report a significantly lower overall SDQ difficulty score than the control group at T3.
H3) The intervention group will report a significantly lower SDQ impact score than the control group at T3.

We also developed the following secondary hypotheses:

H4–7) Compared to the control group, the intervention group will report significantly lower scores in terms of conduct problems (H4), hyperactivity/inattention (H5), and peer relationship problems (H6), and higher levels of prosocial behavior (H7) at T3.
H8) The intervention group will report significantly higher levels of quality of life than the control group at T3.
H9–11) The intervention group will report significantly less frequent headache, abdominal pain, and nausea (H9), less body image dissatisfaction (H10), and fewer sleep problems (H11) than the control group at T3.
H12–14) Compared to the control group, the intervention group will report significantly higher scores in terms of frequency of communicating needs (H12), setting boundaries (H13), and connecting with adults (H14) at T3.
H15) The intervention group will report significantly less school non-attendance than the control group (H15) at T3.

## 1 Methods

We have created an Open Science Framework project (https://osf.io/wusjk/?view_only=6fbd7713834a4580bff061b5d92f7495), which contains study-relevant documents, such as the measures document and codebook, with both English and Danish language versions of the items, power simulation code, the intervention white paper (detailing the intervention content), and additional methodological information.

### 1.1 Eligibility criteria and sample size

To be eligible to participate, parents must have at least one custodial child between the ages of 3 and 17, as well as an electronic device (phone, tablet, or computer) with access to the internet. Parents, and their children, must be able to read and understand Danish, as all materials are provided in Danish only.

We simulated the data structure and assumed moments (mean difference and outcome pooled standard deviation) to determine the minimum sample size for this study using R, version 4.4.1. The sample size was calculated with reference to the primary hypothesis (H1), assuming a power of 90 % and an alpha level of 0.05. The estimate of the outcome pooled standard deviation was based on the post-test results from Pelleboer-Gunnink et al.'s (2015) study of a school-based intervention for children of divorce, which also used the emotional symptoms subscale from the SDQ as a primary outcome. We expected a small to moderate effect, as evidenced by a raw mean difference of 0.40 in the SDQ emotional subscale score between the intervention and control group. The test of relevance is a cross-sectional multi-level model, with children nested within parent. We used a generalized estimating equation (GEE), as these make fewer distributional assumptions and yield more robust standard errors. With these parameters, the simulation suggested that roughly 700 clusters (parents with children) would be needed. Enrollment into the study began on May 1st, 2023, and will close when the target sample is reached or on August 31st, 2024, whichever comes first.

### 1.2 Ethical approval and protocol registration

All procedures are in accordance with the ethical standards of the institutional and national research committee and with the 1964 Declaration of Helsinki and its later amendments or comparable ethical standards. We have received ethical approval from the University of Copenhagen Research Ethics Committee for Science and Health (case number 504–0290/21–5000), and from the Danish Data Protections Agency (case number 514–0699/22–3000). The study is exempt from further ethical evaluations following the rules and regulations as set forth by the Scientific Ethical Committees of Denmark (i.e., national ethics approval was not required). The protocol was registered with clinicaltrials.gov prior to data collection (ClinicalTrials.gov Identifier: NCT05760820).

### 1.3 Procedure

**Fig. 1:**
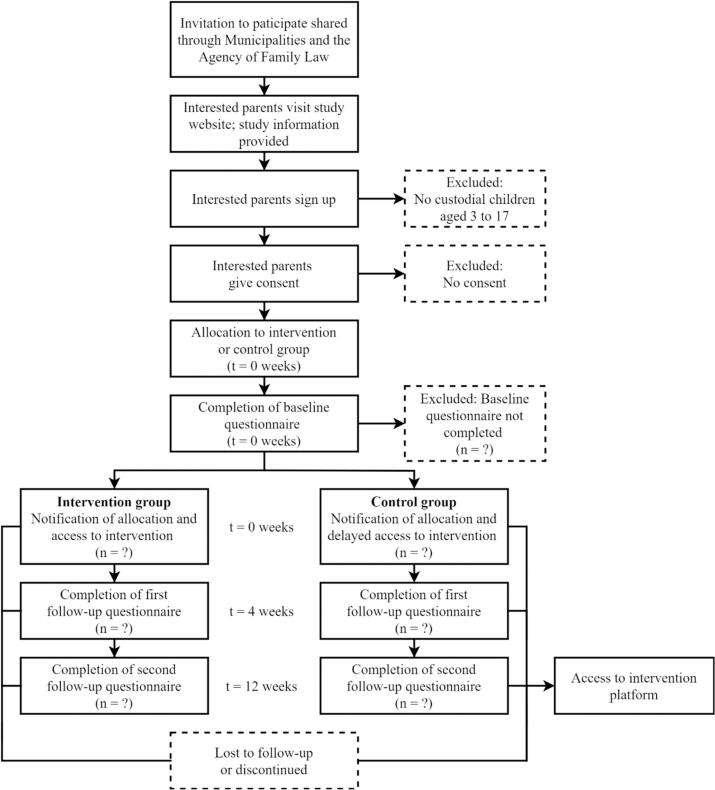
CONSORT diagram.

The SES NXT trial is designed as a randomized controlled, parallel-group, superiority trial that compares a waitlist control group with the digital SES NXT intervention program. The primary endpoint is 12 weeks post-baseline (T3). Fig. 1 presents the CONSORT diagram. We elect to collect data from participants at T1, T2, and T3, for the following reasons: 1) we want to see whether there are short-term positive effects of using the intervention, and we believe that 4 weeks (T2) is sufficient time for participants to access and engage with the intervention, 2) in a previous study of an adult divorce intervention, we found that improvements were largest at the 12-week (T3) time point and plateaued at subsequent time points (i.e., Hald et al., 2020a), and 3) we received push-back from grant reviewers, indicating that longer follow-up times were ethically unacceptable, as they would delay access to the intervention for those in the control group.

Families are recruited through Danish municipalities and the Agency of Family Law; municipal officials distribute informational fliers through the school/daycare online parent portals every 3 months or refer the families directly to the sign-up website. Physical posters and postcards are hung in high-foot traffic areas within the schools and daycares. Moreover, caseworkers can refer clients to the study by sharing the informational fliers. Interested parents access a website that provides information about the study and the study sign-up sheet. Specifically, they are informed about the structure of the study (surveys at baseline, 4 and 12 weeks, the use of a wait-list control group) and the associated incentive structure (movie tickets for survey and module completion; see below), as well as about efforts taken to protect their data and their rights as a participant (e.g., voluntary participation, can withdraw from the study at any time). Moreover, they are provided with contact information for the study responsible, in case of any questions or issues, and contact information for technical support, in case of issues accessing the intervention.

In the sign-up form, they are asked to indicate whether they are the custodial parent of one or more children aged 3–17 years and to provide the name, age, and contact information for each child. Parents provide their own e-mail and/or phone number, or that of their children. Consent is collected at the end of the sign-up form, with a check box. Custodial parents consent to study participation on behalf of themselves and their children; only the parents who sign the children up for the project provide consent. Given the online nature of the study, we are unable to obtain child assent. However, we advise the parents to talk to their child about study participation and use of the digital intervention.

Families are randomized to either the intervention or the wait-list control group. Randomization is performed electronically, with a random number generator, and randomization occurs at the family level, such that parents and children are randomized to the same group, at a 1:1 allocation ratio. Participants (i.e., parents and children aged 11–17) are sent e-mails and/or text messages containing links to the online T1 survey, and upon completion of the T1 survey, they are informed of their group allocation. Intervention group participants receive access to the intervention immediately. Access to SES NXT is provided to the specific child, for whom the T1 survey is completed; that is, if a mother completes a survey for “Child 1”, but not “Child 2”, then only “Child 1” receives access to SES NXT. User accounts are automatically generated for each child that is signed up.

After signing up, parents receive an e-mail and/or text message with a link to a short survey about themselves. Parents also receive individual links via e-mail and/or text message to questionnaires for each child aged 3–10 years. Youths aged 11–17 years receive links via e-mail and/or text message to surveys for them to complete on their own; in cases, where children do not have their own e-mail address or mobile phone number, parents may list theirs and share the survey with the youth. Upon completion of the baseline (T1) questionnaire, participants receive follow-up questionnaires 4 weeks (T2) and 12 weeks post-baseline (T3). Even if participants do not complete the T2 survey they still receive the T3 survey. Table 1 provides an overview of the scales completed at each timepoint. Participants in the control group receive access to SES NXT after T3, regardless of whether they respond to the T2 and T3 surveys.

**Table 1:** Overview of measurement.

	Parent	Age 3	Age 4–10	Age 11–17
	Self-report	Parent report	Parent report	Self-report
Socio-demographic questions and relationship/divorce questions	T1			
Divorce conflict (Hald et al., 2020b)	T1, T2, T3			
Depression symptoms (PHQ-2; Kroenke et al., 2003)	T1, T2, T3			
Anxiety symptoms (GAD-2; Kroenke et al., 2007)	T1, T2, T3			
Strength and Difficulties Questionnaire (Goodman et al., 1998)		T1, T2, T3	T1, T2, T3	T1, T2, T3
Physical health: Somatization (SDQ, item 3), body dissatisfaction, sleep quality		T1, T2, T3	T1, T2, T3	T1, T2, T3
School and daycare attendance (Heyne et al., 2020)		T1, T2, T3	T1, T2, T3	T1, T2, T3
The Quality of My Life Questionnaire (Gong et al., 2007)				T1, T2, T3
Perceptions of parental conflict (Hald et al., 2020b)				T1, T2, T3
Platform focus: Communicating needs, setting boundaries, and connecting with adults			T1, T2, T3 (age 6–10)	T1, T2, T3
Support services		T1	T1	T1

Note. T1 = baseline, T2 = follow-up at 4 weeks post-baseline, T3 = follow-up at 12 weeks post-baseline. The primary and secondary hypotheses specify the child-related questions as outcomes; parent self-report is used as covariates.

To optimize participant retention, we implement two strategies: 1) All participants receive reminder e-mails and/or text messages to complete the questionnaires two and five days after the survey link is sent out. 2) We provide two movie tickets (value 180 DKK/24 EUR) for active participation. Specifically, participants in the control group earn one movie ticket for completing two surveys and one additional movie ticket for completing all three surveys. Participants in the intervention group earn one movie ticket for completing two surveys and three SES NXT modules. They can earn an additional movie ticket for completing all three surveys and three modules on the intervention platform. The tickets are earned individually, for each child.

### 1.4 Intervention description

SES NXT is a digital intervention for children and youth aged 3–17 years, who experience parental relationship dissolution. The intervention includes various elements such as text, videos, motion graphics, and voice-overs, and highly interactional content such as digital activities and exercises. SES NXT is designed to be adaptive to the youth's age. The intervention has four age categories correspond to the Danish daycare and school system, with 3–5-year-olds attending daycare and kindergarten, ages 6–8 years representing pre-school and start of primary education, ages 9–12 years representing primary education and start of lower secondary education, and ages 13–17 years representing lower secondary education and start of secondary education (e.g., high school). The format of the intervention was adapted to reflect the cognitive, social, emotional, and digital competencies of each of those age groups. Specifically, the intervention for children aged 3–5 consists of videos with speak that the parent and child can watch together and contain dialogue questions for the parent to initiate conversations with the child about the theme in question. Youth ages 6–8 are able to manage a digital tool on their own (with less parental guidance), but may not be able to read; therefore, the intervention consists of videos with speak and some short texts at a low reading level. For youth aged 9–12 and 13–17, the intervention consists of videos, texts, and interactive content, reflecting their greater reading level and digital competencies. However, the interventions for the two age groups differ with respect to their level of abstraction. Moreover, videos that contain other children (i.e., “peers”) present children in the same age range as the participants (e.g., 9–12-year-olds watch videos of other 9–12-year-olds).

The intervention addresses key factors associated with adjustment to parental relationship dissolution, such as perceived support (Sorek, 2020), coping skills (O'Hara et al., 2019; Sandler et al., 2000), child-parent relations and communication (Gunter Castleton, 2020; Kunz, 2001; O'Hara et al., 2019; Wal et al., 2021), and the interpretation and meaning made of the relationship dissolution (e.g., loss, gain, change, healing) (Brand et al., 2017; Venta and Walker, 2021). Table 2 provides an overview of the modules included in the intervention for each age group. Each module comprises a theme identified in the literature as central to youths experiencing parental relationship dissolution (e.g., living in two homes, bonus families, and parental conflict). Most of the content in the intervention is covered across the age groups, though in age-appropriate ways. An example of this is the module(s) concerning emotions (i.e., “Feelings” for 3–5-year-olds and 6–8-year-olds and “Understand your feelings” for 9–12-year-olds and 13–17-year-olds), which covers the various emotions that one might experience in response to a parental relationship dissolution. Different emotions are described: sadness and longing, guilt, joy, fear and insecurity, anger. For the two older age groups, the text for sadness and longing is: “Sadness and longing are the best! Because it shows you that someone or something means a lot to you. In other words, it's a sign of love. But... isn't it bad to be sad? No, actually not. It can be tough and hard, but if you were never sad, it would actually be a little sad.” The texts are (often) the same for the 9–12- and 13–17-year-olds and they are introduced to an understanding of emotions as good, even though they can be difficult to experience.

**Table 2:** Intervention content overview, by age group in the intervention.

Theme	Age 3–5	Age 6–8	Age 9–12	Age 13–17
*Family Constellations*				
My Family	X			
The Bonus Family		X	X	X
*Practical Matters*				
Changeover Day	X			
Living in Two Places		X	X	X
Packing Your Bag		X	X	X
*Emotional Aspects of Parental Divorce*				
Missing Someone Means You Care	X			
Feelings	X	X		
Understand Your Feelings			X	X
When It has Just Happened		X	X	X
Tell Your Story			X	X
*Agency*				
Find an Important Adult		X	X	X
Learn To Say Yes and No		X	X	X
My Parents Are Not Getting Along		X	X	X
My Rights			X	X

Building on the example, the theme of longing is also addressed for the 3–8-year-olds. Here, the text is: “Everyone sometimes misses someone they care about. Both children and adults. To miss means thinking about someone who isn't there. Someone you care about. Maybe you can feel it in your stomach? Maybe you become a little sad? It could also be that you're looking forward to seeing the person you miss. That's totally okay.” Children in the 3–5 and 6–8 age groups are introduced to the concept of “longing”, a common emotional response, and are made to understand that they can feel this way when their parents have separated. In essence, for these modules, the intervention seeks to help the younger children to mentalize various feelings, while the older youth are challenged to understand emotions at a higher level of abstraction, as they must recognize that what feels hard may also be important.

The guiding principles interwoven into the digital modules are: 1) *The mind is social* (Tomm, 1984): Human behavior, emotions, and cognitions are situated in a social context. 2) *Re-connection* (Perry, 2013): When children experience significant life changes, connections with existing values, hopes, or dreams may change. The child is guided to re-connect with these in the same or in an altered form, depending on new life circumstances. 3) *Agency* (White, 2008): The experience of and belief in the ability to influence the world around oneself and not only be the passive recipient of what happens around oneself. 4) *Appropriate disturbance* (Schjødt and Ekeland, 2008): An ‘appropriate disturbance’ typically refers to a disruption or challenge that is constructive and suitable within a specific context. This type of disturbance is designed to evoke thought, encourage growth, or create necessary change without causing harm or undue stress. 5) *Normalization* (White, 2008): When youths face parental relationship dissolution, they may feel they have failed or done something wrong, and think they are alone in these difficult emotions. Normalizing these feelings by showing them they are not alone can make them less distressing and intrusive. 6) *Mentalization* (Aasen and Fonagy, 2021): The ability to understand mental states of oneself and other.

The intervention can be accessed at any time during the trial by logging into the intervention website from a mobile smartphone, tablet, or computer. Given that divorce is a heterogeneous process, and the experience of divorce is different for each individual, participants choose the modules that are most relevant to them. The modules can be completed in any order, as little or much as desired, and repeated as needed.

### 1.5 Measures

All questions are completed at baseline (T1), 4 weeks post-baseline (T2), and 12 weeks post-baseline (T3); parents complete questionnaires on behalf of their children aged 3–10, while youth aged 11–17 complete the questionnaires themselves. Table 1 provides an overview of the measurements in the study.

The Strength and Difficulty Questionnaire (SDQ; Goodman, 1997; Goodman et al., 1998) is used to measure emotional, behavioral, and social difficulties. The SDQ consists of a self-report version (from age 11) and proxy report version for parents, both including 25 items rated on a 3-point scale (i.e., 0 = “not true”, 1 = “somewhat true”, and 2 = “certainly true”). The items are divided into five 5-item subscales that generate a score for: 1) Emotional problems, 2) Conduct problems, 3) Hyperactivity/inattention, 4) Peer relationships problem, and 5) Prosocial behavior. Scores for each of the subscales can range from 0 to 10. A total SDQ difficulty score is calculated by summing up the difficulties across the four problem areas (not including prosocial behavior); scores range from 0 to 40, with higher scores indicating more mental well-being related difficulties. The extended version of the SDQ contains an impact scale that asks questions about child distress and interference of problems with home life, friendships, classroom learning, and leisure activities. The SDQ impact scale consists of 5 items, rated on a 4-point scale (i.e., 0 = “not at all”, 1 = “only a little”, 2 = “a medium amount”, and 3 = “a great deal”). The items are summed, and the resultant score can range from 0 to 10, with higher scores indicating greater impact of problems on daily life. The SDQ is a well-established and widely used measure that has shown good psychometric properties in a Danish sample (Arnfred et al., 2019; Niclasen et al., 2012). In this study, the SDQ-Emotional subscale, the SDQ-Total scale, and the SDQ-Impact scale serve as primary outcomes (Hypothesis 1, 2, and 3), while the remaining 4 subscale scores (conduct problems, hyperactivity/inattention, peer relationships problems, and prosocial behavior) are secondary outcomes (Hypothesis 4–7).

The Quality of My Life Questionnaire (QoML; Gong et al., 2007) is used to assess quality of life (QoL). The questionnaire consists of three items: two visual analog scales (VAS) and a categorical measure of change in QoL. The QoML asks “Overall, my life is . . .”. Participants record their responses on a digital VAS for each question stem, which ranges from “the worst” (0) to “the best” (100), with higher scores suggesting better QoL. The questions are only completed by children aged 11 to 17. The QoML serves as a secondary outcome (Hypothesis 8). Previous research established convergent validity in a sample of children living with a chronic health condition (Gong et al., 2007) but the scale has not been validated within a Danish sample.

Physical health is assessed across three domains: somatization, body size perceptions, and sleep quality. Somatization is indicated by one question from the Strength and Difficulties questionnaires, which assesses whether children experience headaches, stomach aches, or nausea (item 3 of the SDQ). This item has previously been used in a national cohort investigation of children's well-being in Denmark (Ottosen et al., 2022). We assessed perceptions of body size, with responses ranging from “much too thin” (1) to “much too fat” (5). This item was drawn from The Health Behaviour in School-aged Children (HBSC) study (Currie et al., 2014), to reflect body image dissatisfaction, a potential predictor of physical and mental health (Currie et al., 2014). Sleep quality is assessed by asking whether the child has had difficulties falling asleep during the last month. This item was also drawn from the HBSC study (Currie et al., 2014, section 5.10). Participants are provided with the following response options: “never” (1), “a couple of times” (2), “almost every week” (3), “more than once a week” (4), and “almost every day” (5). These three items serve separately as secondary outcomes (Hypothesis 9–11). Of note, Denmark is a participating member of the The Health Behaviour in School-aged Children (HBSC) study and thus, the items have been employed in national surveys of school-aged children, aged 11, 13, and 15 years (Madsen et al., 2023).

Participants also respond to questions that tap into key domains targeted by the intervention. These domains concern 1) communicating needs, 2) setting boundaries, and 3) connecting with an adult. Each domain contains three questions developed specifically for this study. The item content for domains 1) communicating needs and 3) connecting with an adult is based on the concepts of instrumental, emotional, and informational support (Taylor, 2011). For the communication needs and setting boundaries domain, the responses are rated on a 4-point scale (e.g., 0 = “No and I haven't needed help”, 1 = “No, even though I have needed help”, 2 = “Yes, one time”, and 3 = “yes, multiple times”). For the connection with an adult domain, responses are rated on a 5-point scale (ranging from 1 = ranging from “very difficult” to 5 = “very easy”). Items for each domain are averaged to create a mean score, and higher scores indicate higher frequency of communication needs, greater frequency of setting boundaries, and lower difficulty in connecting with an adult. For the communicating needs domain, participants report if they have asked for instrumental support (e.g., asked for help with money), emotional support (e.g., told someone that you are upset) and informational support (e.g., asked for advice). For the setting boundaries domain, participants are asked whether they have spoken up if parents have spoken badly about each other, have had a verbal argument in front of them, and if the parents have tried sending each other messages through them. For the connecting with an adult domain, participants are asked how easy or difficult it has been for them to seek instrumental support, emotional support, or informational support. These items were developed specifically for this study by the first and last author, and thus, no psychometric information exists for these items. These three domains serve as secondary outcomes (Hypothesis 12–14).

School non-attendance is measured with one item that examines whether and how many days the child has been absent from school or day care, over the last 4 weeks (for T1 and the T2) or 8 weeks (for T3). Participants respond with the number of days, ranging from 0 to 20 days for T1 and the T2 (i.e., 4-week school- or daycare-period), and 0 to 40 for the T3 (i.e., 8-week school- or daycare-period). Participants are also able to respond that they do not know or that the question is not relevant to them (in cases of vacations or the child not attending school/daycare). The item for school non-attendance follows the construct guidelines proposed by Heyne et al. (2020). A percentage will be calculated for full days of absence, such that higher percentages indicate higher school non-attendance; this variable serves as a secondary outcome (Hypothesis 15).

Degree of conflict is reported by parents and youths (11 to 17 years) using the 6-item self-report Divorce Conflict Scale, developed and validated within a Danish sample (DCS; Hald et al., 2020b). Participants respond to the items using a 4 to 6-point rating scale (response options vary by item stem). Items are summed together to create a score that can range from 0 to 27, such that higher scores indicate greater conflict. This variable serves as an exploratory outcome, as we expect that parents may reduce their conflict in response to 1) exposure to the content of the child intervention and 2) because the child fares better, as child well-being and co-parenting can be a point of conflict for parents (e.g., Johnston, 1994; Hald et al., 2020b).

### 1.6 Statistical analyses

Due to the nesting of participants within family units, all analyses are conducted within a multilevel modelling framework, accounting for the interdependence in the data (children nested within parent). Attrition bias analyses will be conducted, comparing participants who only completed T1 to those who remained in the study after T1 (i.e. completed T2 and/or T3 assessment), using multilevel logistic regression, with drop-out as the outcome and baseline sociodemographic and psychological variables as predictors.

To examine the effectiveness of the intervention, multilevel regressions will be conducted to examine group differences on the primary outcomes: SDQ-Emotional (H1), SDQ-Total (H2), and SDQ-Impact (H3) at T3. These analyses will be based on the intention-to-treat principle (Gupta, 2011). Multiple imputations using chained equations as implemented in the R package mice will be applied to handle missing data. The regression models will be specified to allow for a random intercept, as well as for interdependence due to nesting of children within parent participants, using a generalized equating equation. Thus, in these analyses, participant responses are the unit of analysis. Group membership (i.e., SES NXT group (1) vs. control group (0)) will be entered as a categorical predictor; we will also include potential covariates, including participants' gender and age, participants' baseline score on the outcome, and parental educational level, income, and depression/anxiety symptoms. Following examination of the primary outcomes, the same analyses will be conducted with respect to the secondary outcomes. For all analyses, a *p*-value of less than 0.05 will be used as the threshold for statistical significance, since this value was used as the acceptable risk of type I error in our sample size estimation (see ‘Sample size’). Additional follow-up analyses will be executed to examine whether there is a differential change over time for the two groups, as reflected in a time-by-group interaction effect and, for the intervention group, to examine differences in the outcomes based on intervention age group and number of modules completed in the intervention.

## 2 Discussion

Developing an effective intervention for youths experiencing parental relationship dissolution is of critical importance, as a great number of youths experience negative short- and long-term consequences relating to a breakup between their parents. The digital SES NXT intervention covers a wide range of topics in a media-rich environment, is tailored to the age of the child, and is scalable. Strengths of the study include the use of sound psychometric measures, and the inclusion of family units (i.e., parent and children), as opposed to individual children, in a study with an RCT design. Potential limitations include issues relating to the completion of surveys by 11- to 17-year-olds, who may not receive the links to the surveys in cases of parents providing their own e-mail address and phone number, or who may simply elect to not complete the survey. Moreover, the online nature of the study limits us from assuring that children aged 11 to 17 complete the survey in privacy. It may be that some of the children complete the survey together with their parent; the parental presence may influence the child's responses in various ways such that the responses do not reflect their true feelings and thoughts. For instance, it may be that children alter their responses to please their parents, to not worry them, or to gain additional attention from them. Technical breakdowns and outages may also occur, such that participants cannot access the intervention. We will regularly check and monitor the technical systems to ensure that the intervention website does not crash or become unavailable and that the API (application programming interface) that connects the various electronic systems works correctly, to minimize the risk of breakdowns. Moreover, there will be a support phone number and e-mail address that participants can contact to report technical issues and receive help. Furthermore, there may be self-selection into the study, as many studies have found that those with better health, higher education and income, and of female gender are more likely to seek treatment (Wellstead, 2011) and to participate in research studies (Søgaard et al., 2004).

Future research should seek to compare this digital intervention with a face-to-face intervention to see whether the digital intervention is more (or less) efficacious in improving child mental health and well-being post parental relationship dissolution; moreover, it would be interesting to see whether the combination of face-to-face and digital intervention yield additional improvements, beyond that gained from just the face-to-face or digital intervention separately. Moreover, in the current work, we assess the effects of the digital intervention at 3-month follow-up. Future research would benefit from extending this timeline, as it would be useful to see whether the effectiveness of the digital intervention yield improvements beyond the initial three months assessed in this study. In sum, the present study will contribute to the extant knowledge about effectiveness of digital health technologies and interventions for youths experiencing parental relationship dissolution and potentially contribute to a cost-effective evidence-based scalable psychological help for a population who needs it.

## Plans to give access to the full protocol, participant level-data and statistical code

We are unable to share individual data in any form but will happily share upon reasonable request. Please contact Gert Martin Hald via e-mail (ghald@sund.ku.dk) to make such a request. The statistical code associated with each manuscript will be submitted as part of supplemental materials and/or shared on the project's Open Science Framework page.

## Registration in trials

The study has been registered with ClinicalTrials.gov, Identifier: NCT05760820

## Funding

The intervention development was supported by a grant provided by the 10.13039/501100009902Egmont Foundation to Samarbejde Efter Skilsmisse ApS. The research project received financial support from 10.13039/501100005860Helsefonden, under grant no. 22-B-0105, and from 10.13039/100012774Innovation Fund Denmark, under grant no. 1045-00042B.

## Declaration of competing interest

The authors declare the following financial interests/personal relationships which may be considered as potential competing interests: Gert Martin Hald reports financial support was provided by Health Foundation and Innovation Fund Denmark. Daniel B. Johnsen reports a relationship with Samarbejde efter Skilsmisse, ApS that includes: employment. Martin Skriver reports a relationship with Samarbejde efter Skilsmisse, ApS that includes: employment. Soeren Sander reports a relationship with Samarbejde efter Skilsmisse, ApS that includes: equity or stocks. Gert Martin Hald reports a relationship with Samarbejde efter Skilsmisse, ApS that includes: equity or stocks. If there are other authors, they declare that they have no known competing financial interests or personal relationships that could have appeared to influence the work reported in this paper.

For due diligence, we would like to declare that 2 of the co-authors (Gert Martin Hald and Søren Sander) hold the commercial license and intellectual property rights to the SES NXT intervention through ‘Samarbejde Efter Skilsmisse ApS’, but neither have influence over or decision power in the analytical and statistical approaches taken in the RCT trials of the intervention.
